# Mapping discourse coalitions in the minimum unit pricing for alcohol debate: a discourse network analysis of UK newspaper coverage

**DOI:** 10.1111/add.14514

**Published:** 2019-01-04

**Authors:** Gillian Fergie, Philip Leifeld, Ben Hawkins, Shona Hilton

**Affiliations:** ^1^ MRC/CSO Social and Public Health Sciences Unit University of Glasgow Glasgow UK; ^2^ Department of Government, University of Essex; and School of Social and Political Sciences University of Glasgow Glasgow UK; ^3^ Faculty of Public Health and Policy London School of Hygiene and Tropical Medicine London UK

**Keywords:** Alcohol, health, policy, public health, discourse, networks

## Abstract

**Background and Aims:**

Minimum unit pricing (MUP) for alcohol was introduced in Scotland on 1 May 2018, and is now on the policy agenda in other devolved administrations and at Westminster. Previous research has explored the arguments deployed for and against MUP, but the congruence between actors in the MUP debate has not been sufficiently examined. This study identified and mapped the discourse coalitions that emerged in the UK MUP debate through an analysis of actors’ use of arguments in media coverage of the policy debates.

**Design:**

A sample of print media coverage of MUP was obtained from the LexisNexis newspaper database. The resulting sample was imported into discourse network analysis (DNA) software for coding and subsequent visualization of actor networks.

**Setting:**

United Kingdom.

**Observations:**

A total of 348 articles from eight UK‐wide and three Scottish newspapers from an 18‐month period, ending in November 2012, were analysed.

**Measurements:**

Actors’ arguments were coded to generate structured data for conversion into a weighted actor network where ties represent similarities among actors in terms of arguments in support of or opposition to MUP.

**Findings:**

Two polarized discourse coalitions, Opponents and Proponents of MUP, emerged in media coverage. The Proponents coalition consisted mainly of health advocacy groups, charities, political parties and academic institutions. In the Opponents coalition, the networks were formed of key alcohol manufacturers and economic think‐tanks. While producer organizations were central to the Opponents coalition, some commercial actors were more favourable to MUP, highlighting divisions within the industry overall.

**Conclusions:**

Media coverage of minimum unit pricing (MUP) in Scotland from June 2011 to November 2012 showed alignment between the policy positions of (1) alcohol producers and think‐tanks opposed to MUP; and (2) public health advocates and health charities in favour of the policy. Some alcohol industry actors were supportive of MUP indicating divisions among the industry. Discourse network analysis may be usefully applied to study other highly contested policy issues in health and beyond.

## Introduction

Forms of minimum alcohol pricing have been in place throughout various Scandinavian countries and Canadian provinces for several years, offering governments an opportunity to both raise revenue and stabilize markets 1. The implementation of similar policies, with the explicit aim of reducing consumption and minimizing health harms, has been widely debated since the Scottish Government announced its interest in Minimum Unit Pricing (MUP) for alcohol. Attracting international interest, the policy is now under consideration among various international contexts, including Ireland, Estonia 2 and Australia 3, as well as elsewhere in the United Kingdom. In these contexts, and throughout the United Kingdom, the impact of MUP is heavily anticipated. Following the UK Supreme Court ruling in November 2017 against the Scotch Whisky Association's challenge to the policy, MUP was introduced in Scotland on 1 May 2018 4. This internationally significant policy passed into law in Scotland in June 2012 (the Alcohol (Minimum Pricing) (Scotland) Act). At Westminster, the extension of the policy to England was shelved in 2013, with the Conservative–Liberal Democrat coalition government citing a lack of evidence; it remains under review 5. In Wales, the Public Health (Minimum Price for Alcohol) (Wales) Bill was introduced to the National Assembly in October 2017 and in Northern Ireland the Department of Health have announced a consultation paper on MUP for consideration in 2018 6. The progress of MUP has been highlighted as an illustration of the complexity of the policy process 7, with a range of factors determining the status and traction of the policy across political contexts 8, 9.

Complex policy processes can be conceptualized as networks of political ‘actors’ or stakeholders with an interest in the formation of a particular policy, that form coalitions in order to shape and influence policy debates 10, 11. The Advocacy Coalition Framework (ACF) posits that actors with similar normative policy beliefs can be identified and that these entrenched coalitions compete about policy design over long time‐periods 12. Argumentative discourse analysis suggests that actors influence each other through arguments and position themselves at ‘particular sites of discursive production’, which are referred to as ‘discourse coalitions’ 13. Exploring the discourses produced in a policy debate, the networks of actors that coalesce around particular assemblages of beliefs, and the discourse coalitions that emerge from debates, provides insights into the complex process of policy development.

In relation to MUP, a broad range of actors have been identified as stakeholders in the policy debate. Previous work on media representations of MUP highlights the role of advocates (public health groups, health charities and academics) in making supportive statements about the policy in media coverage of the debate 14. Support for MUP was also evident among wider policy stakeholders, including the police and National Health Service (NHS) 15. Previous research on alcohol policy in general, and MUP in particular, has also highlighted the importance of the alcohol industry as key influencers 16, 17, 18, 19. Direct mechanisms of industry influence include responses to consultations, lobbying activities, widespread media presence and legal challenges 16, 19. More indirect influence by industry through organizations such as think‐tanks 18 and charities 17, as well as through alliances with consumer/civil society groups and engagement in social responsibility activities 19, has also been noted.

Currently, a nuanced picture of the congruence between actors in the MUP debate is lacking. No studies have provided visual representations which reflect the polarization and configuration of actors and organizations involved in the debate. Consequently, the present paper seeks to identify the discourse coalitions active in the alcohol policy debate through actors’ use of arguments in print media coverage of MUP using the method of discourse network analysis 11, 20. By exploring in detail explicit agreement and disagreement between organizations involved in alcohol policy development such analyses can provide opportunities for triangulation of existing evidence on policy influences, offer new insights on the impact of relationships between actors on stated policy positions and help to identify sites for targeted public health advocacy.

## Methods

### Design

The Discourse Network Analysis (DNA) method, which combines qualitative content analysis with network analysis to facilitate the study of policy debates 11, 20, was applied to UK newspaper coverage of the development of MUP during a period of intense debate.

### Data

Eight UK‐wide and three Scottish newspapers (and Sunday counterparts) were searched during a 19‐month period, ending in November 2012, for mentions of Alcohol* OR Booze OR Liquor OR Hooch [in the headline] AND Price OR Pricing or Tax OR Levy [anywhere in text] using the LexisNexis database. The publications were selected to represent three genres of newspaper: ‘serious’/broadsheet, mid‐market and tabloid; and a range of readership profiles in relation to age, social class and political alignment, as in previous studies 21. A full list of publications is available in the Supporting information, Data S1. The search period commenced on 1 May 2011, before the formation of a Scottish National Party (SNP) majority Scottish Government in the Scottish elections held on 5 May 2011 and ended 30 November 2012, after the announcement of the UK Government's consultation on their alcohol strategy. The period covered was selected to include a peak in reporting on the policy identified by Patterson and colleagues 22. The search identified 937 articles. All articles were read to determine whether they met the pre‐defined inclusion criteria: MUP was the main focus of the articles, and the article was a news, commentary or feature piece (readers’ letters were excluded). After exclusions, 348 articles were included.

### Measures

Using Discourse Network Analyzer (DNA), 1Available at: https://github.com/leifeld/dna (accessed September 2018) (Archived at http://www.webcitation.org/74jTxwMZz on 17 December 2018). extracts of newspaper text which featured actors’ arguments on alcohol consumption and MUP were coded as ‘statements’. Each statement was coded for four variables: individual actor's name, organizational association of the actor, the argument referred to by the actor (henceforth called ‘concept’) and a dummy variable for agreement or disagreement by the actor with the concept. This approach follows that developed by Leifeld 20. As only direct quotes and reported speech from actors in the debate are coded, journalistic/editorial comment is not included in the analysis. In total, 1924 statements made by 152 individuals from 94 organizations, relating to 56 concepts, were coded. Following initial coding, a 10% sample of articles was coded blind, inconsistencies in coding were discussed and resolved and refinements made to some concepts. All articles were recoded applying this final coding framework. A table of actors and actor types and list of concepts are provided in the Supporting information, Data S2 and S3.

### Data analysis

DNA was used to convert the structured data into a weighted actor × actor network, where ties and their weights represent similarities among actors in terms of agreement and disagreement over concepts. The tie weight between any two actors was calculated as the number of different concepts they jointly supported or jointly rejected over the course of the 18‐month period, minus the number of concepts on which the actors had diverging opinions, divided by the average number of statements made by both. This is a ‘subtract’ transformation with ‘average activity normalization’ 20. This definition of tie weights measures argumentative similarity in excess of differences of opinion and ensures that only argumentative similarity, but not the rate at which actors issue statements, is considered for the calculation of tie weights. Negative tie weights represent differences in opinion in excess of argumentative similarity, while positive tie weights represent similar argumentative positions in excess of differences in opinion, aggregated over all concepts and the whole time‐period. A threshold value of ≥ 0.4 was determined in an exploratory way and applied to the resulting network to retain only relatively robust argumentative similarity as ties in the network 20.

The actor network was then imported into the network visualization software *visone* to map actors and their coalitions visually. 2Available at: http://www.visone.info/ (accessed September 2018) (Archived at http://www.webcitation.org/74jTjlCmV on 17 December 2018). Actors in the network are represented as nodes and ties (calculated as above) and are represented by linear connections between nodes. Girvan–Newman edge‐betweenness community detection—a common graph clustering algorithm (see 23)—was applied to the network in order to identify discourse coalitions as cohesive subgroups with similar argumentative patterns. The discourse coalitions were highlighted in the network visualizations as blue hyperplanes. Different actor types were highlighted using colours, and the statement frequency of each actor was visualized as the size of the respective node.

In order to check for bias inherent in publications chosen, separate networks were created for each individual newspaper. Broadly, these showed polarized coalitions in line with the complete network (although smaller and hence with less certainty). These networks are available in the Supporting information, Data S4.

Finally, three half‐year time slices of the network were created to track changes in the policy debate over time. For each time slice, bar plots were created for the concepts to indicate how common and how contested each argument was in each phase of the debate.

## Findings

A range of types of actors were represented in media coverage of the debate. The organizational associations of individual actors included political parties, charities, advocacy groups, professional associations, think‐tanks, alcohol manufacturers, retailers and retail associations and licensed traders. All the types of organizations are represented in the main component of the network.

### Proponents and opponents

**Figure 1:**
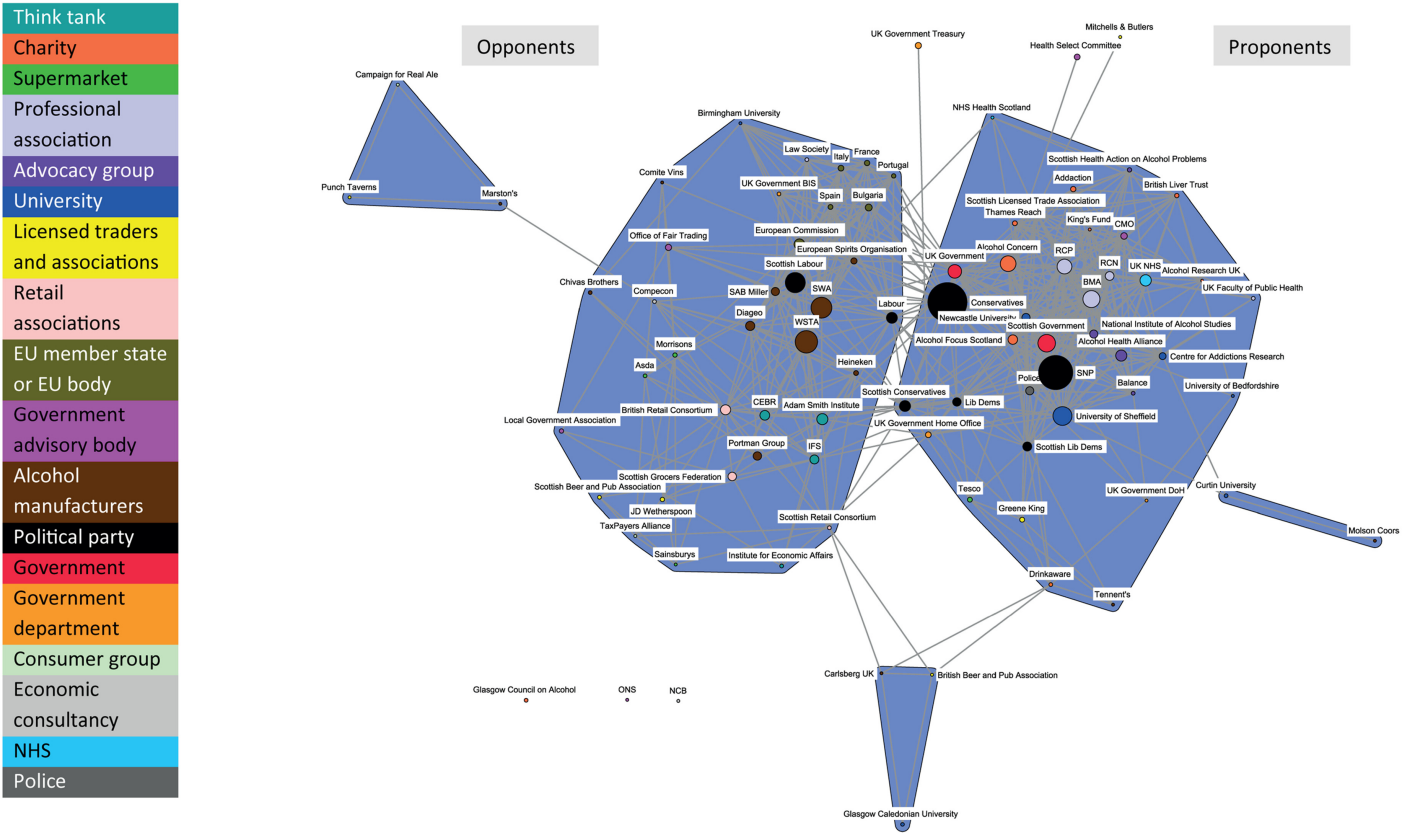
Discourse network for minimum unit pricing (MUP) (organizations)

Two discourse coalitions are evident in the MUP network. Figure 1 shows the coalition of proponents of MUP on the right‐hand side of the network diagram and the coalition of opponents of MUP on the left‐hand side. These discourse coalitions represent similarities among actors in terms of co‐support or co‐rejection of concepts related to MUP.

The ‘Proponents of MUP’ coalition (henceforth referred to as Proponents) includes health charities and advocacy groups, five UK and Scottish government and opposition political parties, some alcohol manufacturers, some licensed trade representatives and most academic institutions (see right side of Fig. 1). Central organizations within this coalition include the SNP and several professional associations (Royal College of Physicians, Royal College of Nursing and British Medical Association). These organizations are drawn together by agreement on a range of concept statements relating to alcohol problem definition, drivers of alcohol harms and MUP as a potential solution; see Table 1 for examples.

**Table 1:** Concepts shared by Proponents.

Concept	Example of statement
Government action is needed on alcohol consumption	‘The government's ongoing failure to tackle the root causes of alcohol misuse means we will see hospital admissions continue to rise in the future.’ Don Shenker (Alcohol Concern), 26 May 2011, *Guardian*
Commercial interests should be limited to protect public health	‘In challenging the implementation of minimum pricing, the Scotch Whisky Association is essentially arguing that the commercial interests of its members should take precedence over the health and wellbeing of the people of Scotland. We need to decide if our society is prepared to limit commercial activity to better protect our health and quality of life, or alternatively, allow powerful corporate interests to dictate health policy.’ Evelyn Gillan (Alcohol Focus Scotland) 26 October 2012, *Guardian*
MUP needed to reduce health harms/deaths related to alcohol	‘Minimum pricing will reduce the harm caused by alcohol misuse and we believe the policy, agreed by Parliament and backed by expert opinion, is the most effective pricing measure and it will save lives.’ Alex Neil (SNP), 29 December 2012, *Daily Record*

MUP = minimum unit pricing.

The ‘Opponents of MUP’ coalition (henceforth referred to as Opponents) consists of some alcohol industry manufacturers, most large supermarkets, alcohol trade organizations, economic think‐tanks, some academic institutions and both Scottish and UK Labour (opposition) parties (see left side of Fig. 1). Central organizations within this discourse coalition include the Scotch Whisky Association and the Wine and Spirit Trade Association. These organizations are drawn together by agreement on conceptions of the alcohol problem, ideological opposition to public health interventions and criticism of the target population for MUP and proposal of alternative solutions to perceived alcohol problems; see Table 2 for examples.

**Table 2:** Concepts shared by Opponents.

Concept	Instance
Alcohol‐related harm is falling/stabilizing	‘It is surprising that hospital admissions have apparently doubled over a period in which alcohol consumption has significantly declined. If the hospital admissions data are robust, they clearly put paid to the argument that measures to reduce overall alcohol consumption are effective in reducing harm.’ David Poley (Portman Group), 27 May 2011, *Daily Mail*
Government action on public health is unnecessary	‘It's regressive and paternalistic, treating people as if they're children to be nannied by the Government.’ Sam Bowman (Adam Smith Institute), *Independent.co.uk*, 14 May 2012
MUP is an unfair cost to consumer and will penalize responsible drinkers	‘It [MUP] is more likely to have a bigger proportionate impact on responsible drinkers who happen to be in low‐income households.’ Andrew Lansley (Health Secretary, Conservative), 18 December 2011, *Independent*
Responsibility deals with industry are effective	‘We have also had significant success with community alcohol partnerships aimed at combating under‐age drinking; the Responsibility Deal is taking a billion units of alcohol out of the market.’ Gavin Partington (Wine and Spirit Trade Association), 30 March 2012, *The Times*.

MUP = minimum unit pricing.

### Network and coalition development

**Figure 2:**
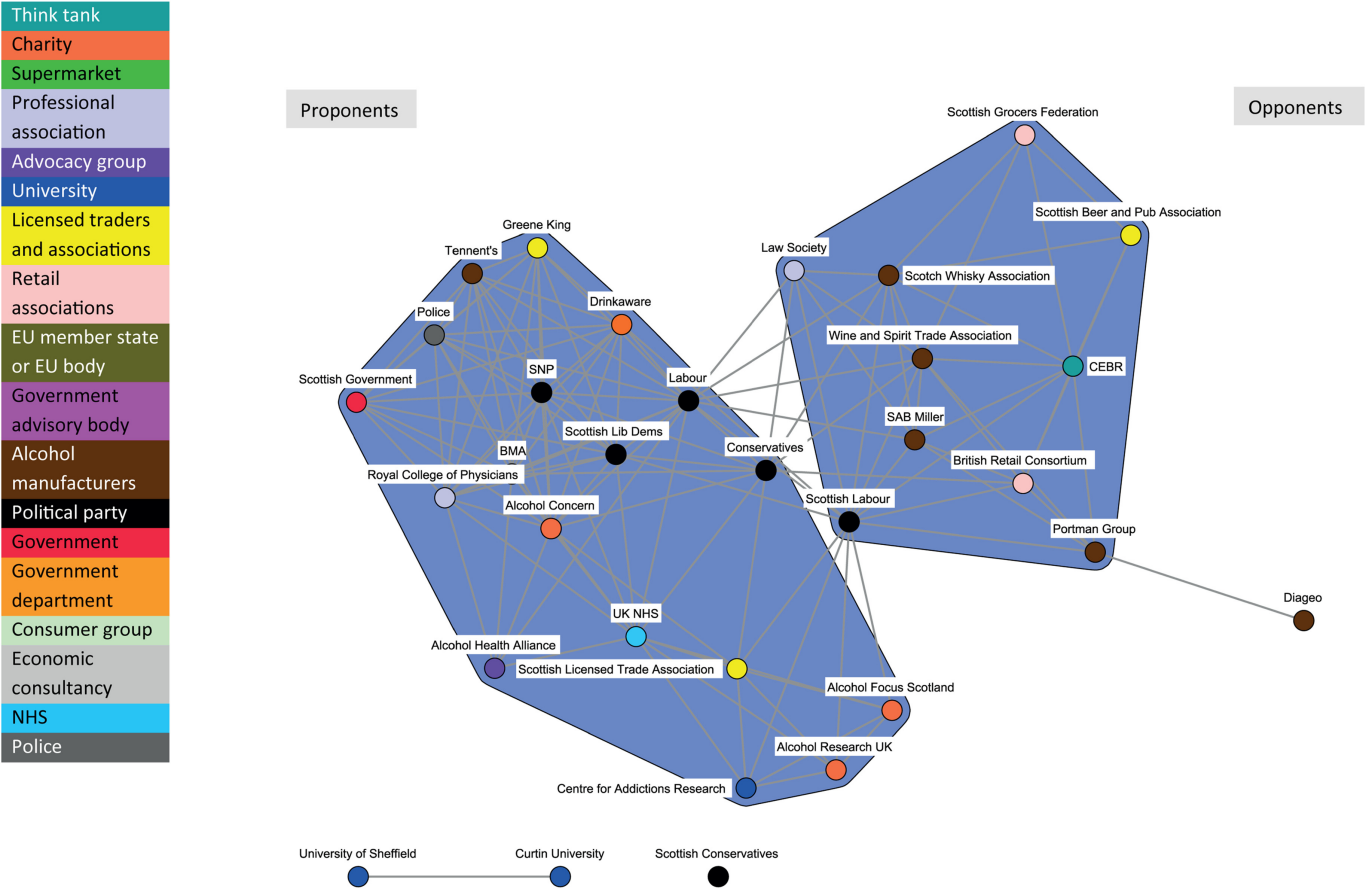
Discourse network for minimum unit pricing (MUP) (organizations): 1 May 2011–30 October 2011

The dynamic nature of policy coalitions is illustrated by comparing Fig. 2 (covering the period from 1 May 2011 to 30 October 2011), Fig. 3 (1 November 2011 to 30 April 2012) and Fig. 4 (1 May 2012 to 30 November 2012).

**Figure 3:**
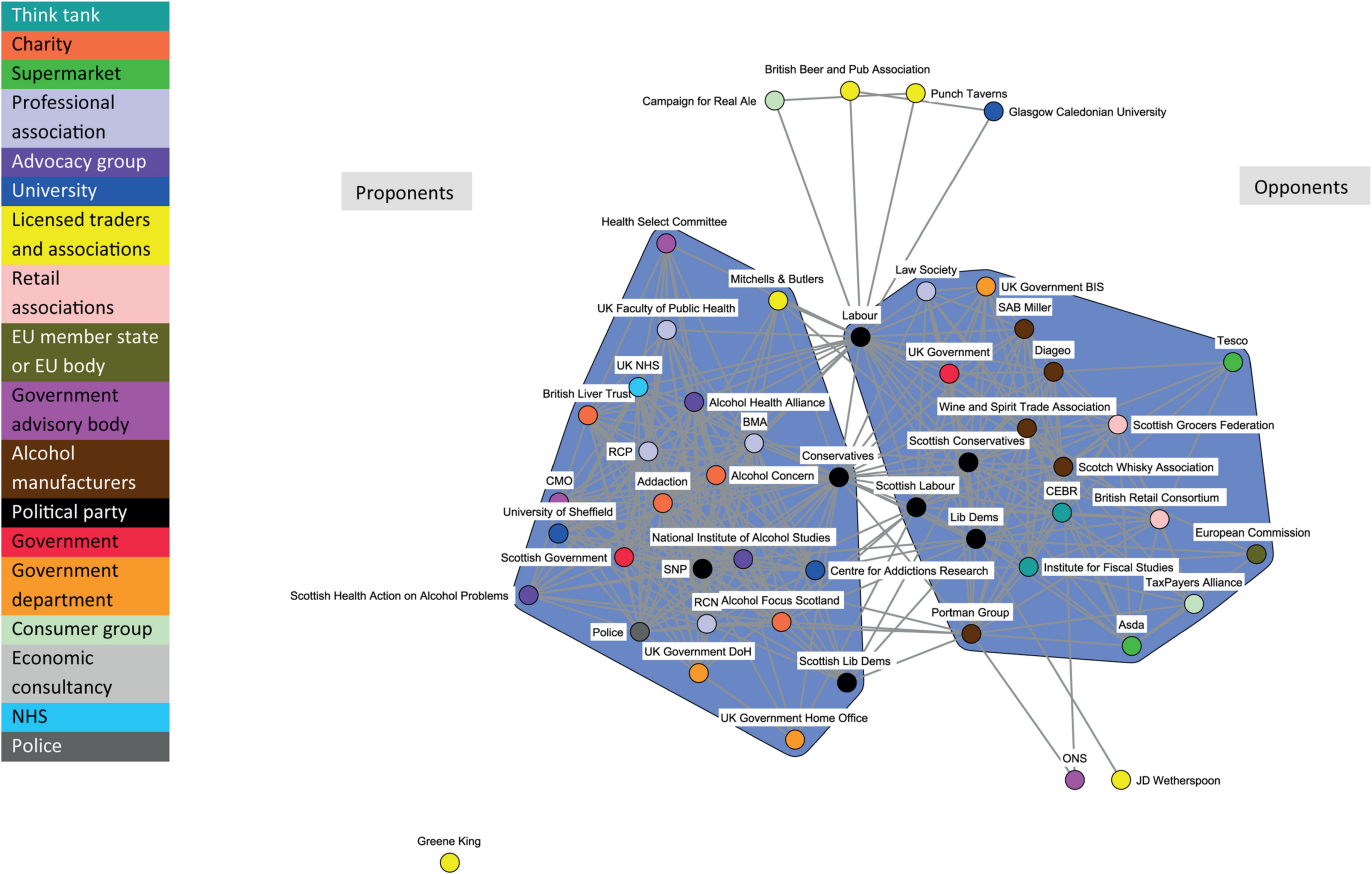
Discourse network for minimum unit pricing (MUP) (organizations): 1 November 2011–30 April 2012

**Figure 4:**
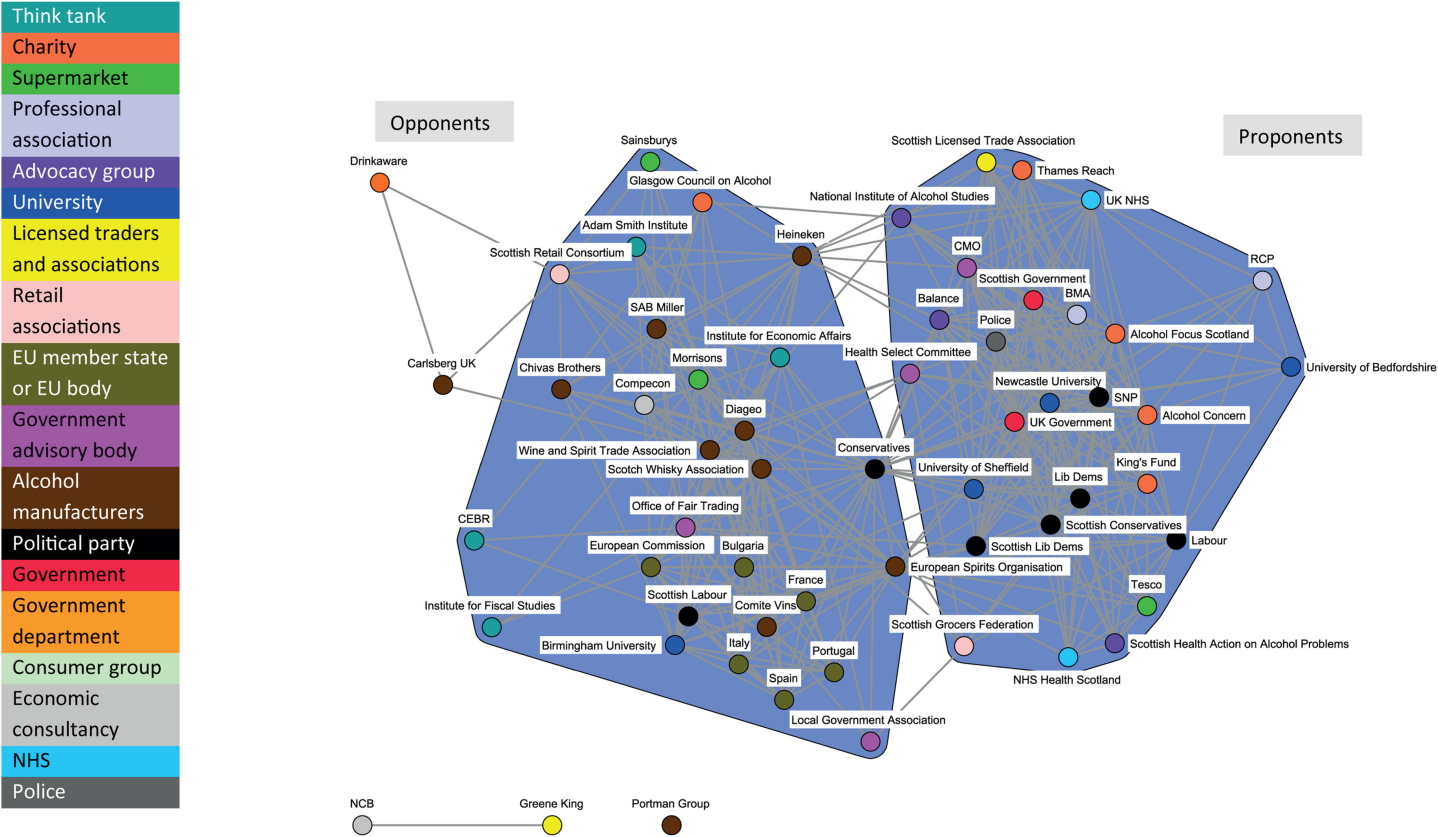
Discourse network for minimum unit pricing (MUP) (organizations): 1 May 2012–30 November 2012

Figure 2 illustrates the relatively small number of actors engaged in the debate during the earliest phase, but indicates clearly the formation of the Proponents and Opponents coalitions. Figure 3 shows an increased number of actors engaging in the policy debate during the second phase, including supermarkets and government advisory bodies. Figure 4 highlights few changes to the network structure during the third phase, with the exception of the UK Conservatives position. They move from the Proponents to the Opponents coalition. This shift is slight, as the node retains a large number of ties across both coalitions; however, this could be an early indication of their future U‐turn on MUP in the UK context.

Bar plots indicating the frequency of concepts referred to, and the frequency of agreement/disagreement, during the early, middle and later phases, show the change over time in the prominence of specific concepts influencing the network.

**Figure 5:**
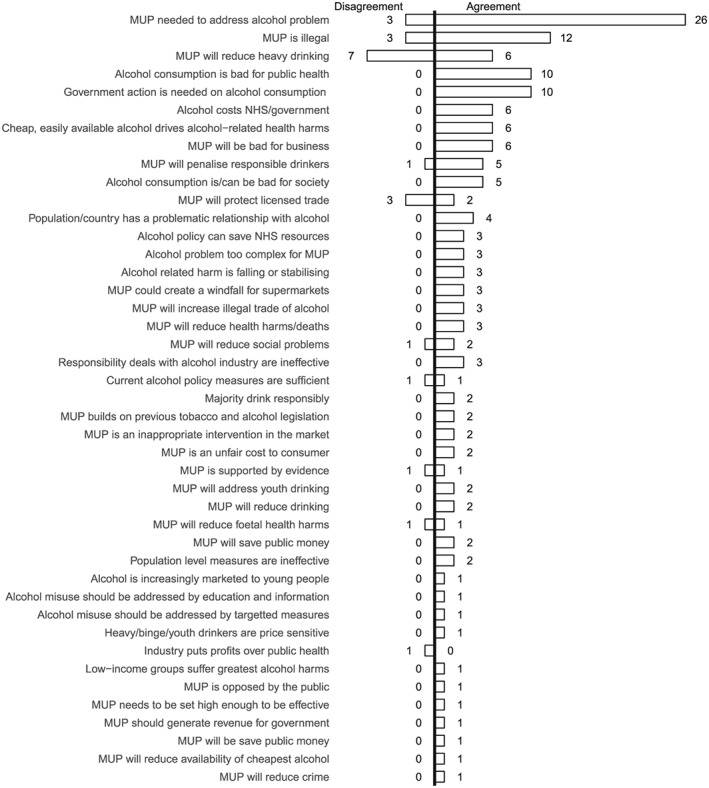
Frequency of reference to concepts: 1 May 2011–30 October 2011

**Figure 6:**
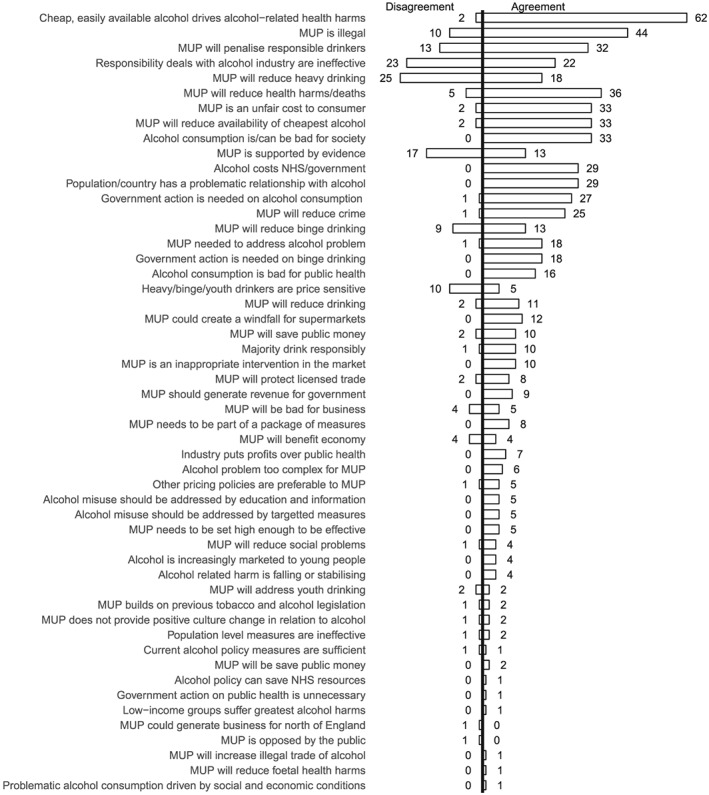
Frequency of reference to concepts: 1 November 2011–30 April 2012

**Figure 7:**
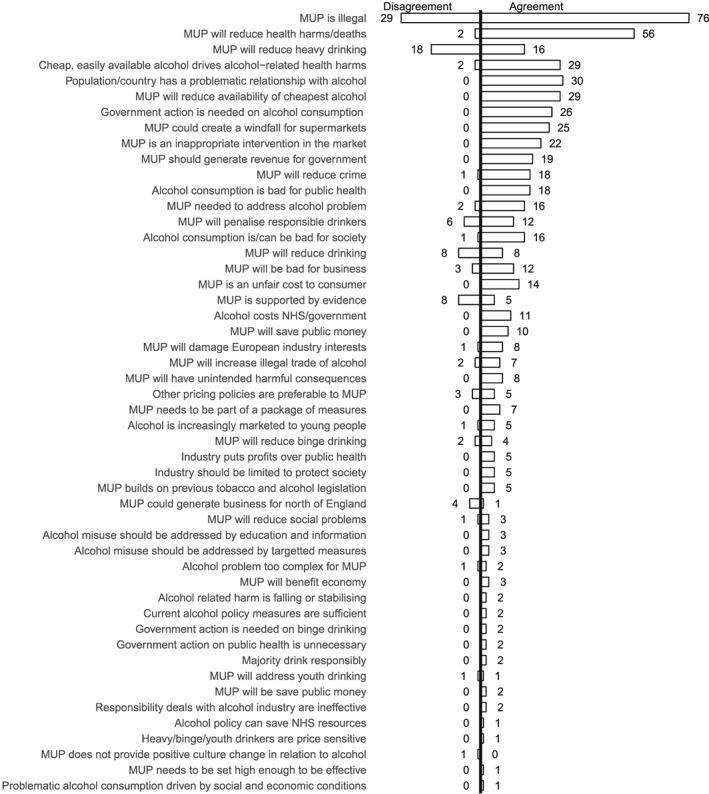
Frequency of reference to concepts: 1 May 2012–30 November 2012

Figure 5 shows that between May and October 2011 the most frequently mentioned concept is around the necessity of MUP to address the alcohol problem, an argument made most often by Proponents. The second most popular concept is ‘MUP is illegal’, an argument which draws together the Opponents coalition. The concept which most divided opinion (equivalent levels of agreement and disagreement) is the potential for MUP to reduce heavy drinking, reflecting the focus on the efficacy of the policy as a targeted population measure. By the middle phase (Fig. 6), this concept remained a focus of contention. Two further polarizing concepts, that responsibility deals with industry are ineffective (23 disagree, 22 agree) and that MUP is supported by evidence MUP (17 disagree, 13 agree) are also evident. By the later phase (Fig. 7), legality and efficacy were the top divisive concepts.

Despite a slight shift in organizational positions within the network, insights from the argumentation of the debate suggest that the key issues during the period remained relatively consistent, with increased division based on perspectives on responsibility deals and the evidence base for MUP evident in the middle phase.

### Political parties and coalition centrality

Figure 1 shows the centrality of political parties as key nodes linking the Opponents and Proponents coalitions. Table 3 provides a count of each political party's total ties in the network, the number of these ties that exist within their primary cluster and a resultant measure that captures the ratio of cluster–external ties over all ties of a given actor (‘external ratio’).

**Table 3:** Political party ties.

Party	Total ties	Ties within cluster	External ratio
Conservatives	33	20	0.39
Labour	24	17	0.29
Liberal Democrats	19	18	0.05
Scottish National Party	24	24	0.00
Scottish Labour	19	18	0.05
Scottish Conservatives	24	13	0.46
Scottish Liberal Democrats	19	18	0.05

The higher the external ratio score, the less central and embedded the party is within its respective coalition. As illustrated in Fig. 1, the Conservatives, Labour and Scottish Conservatives nodes operate across the coalitions, with links to both Proponents and Opponents, making their scores relatively high. In contrast, the SNP score of 0.00 reflects the centrality of the party within the Proponents coalition, with no links to organizations outside the coalition. Scottish Labour also has a low score, as it is embedded within the Opponents coalition. The Liberal Democrats and Scottish Liberal Democrats have low external ratio scores, indicating their embeddedness within the Proponents coalition. None of the political parties inhabit positions at the outer edges of the network or their respective coalitions; rather they are either central to the network or to a particular coalition. The high external ratio score of the UK Conservative Party reflects the number of individual politicians who voiced opposing perspectives on MUP in the media during the course of the debate. This can be further explored through visualization of the network at individual actor, rather than organizational, level.

**Figure 8:**
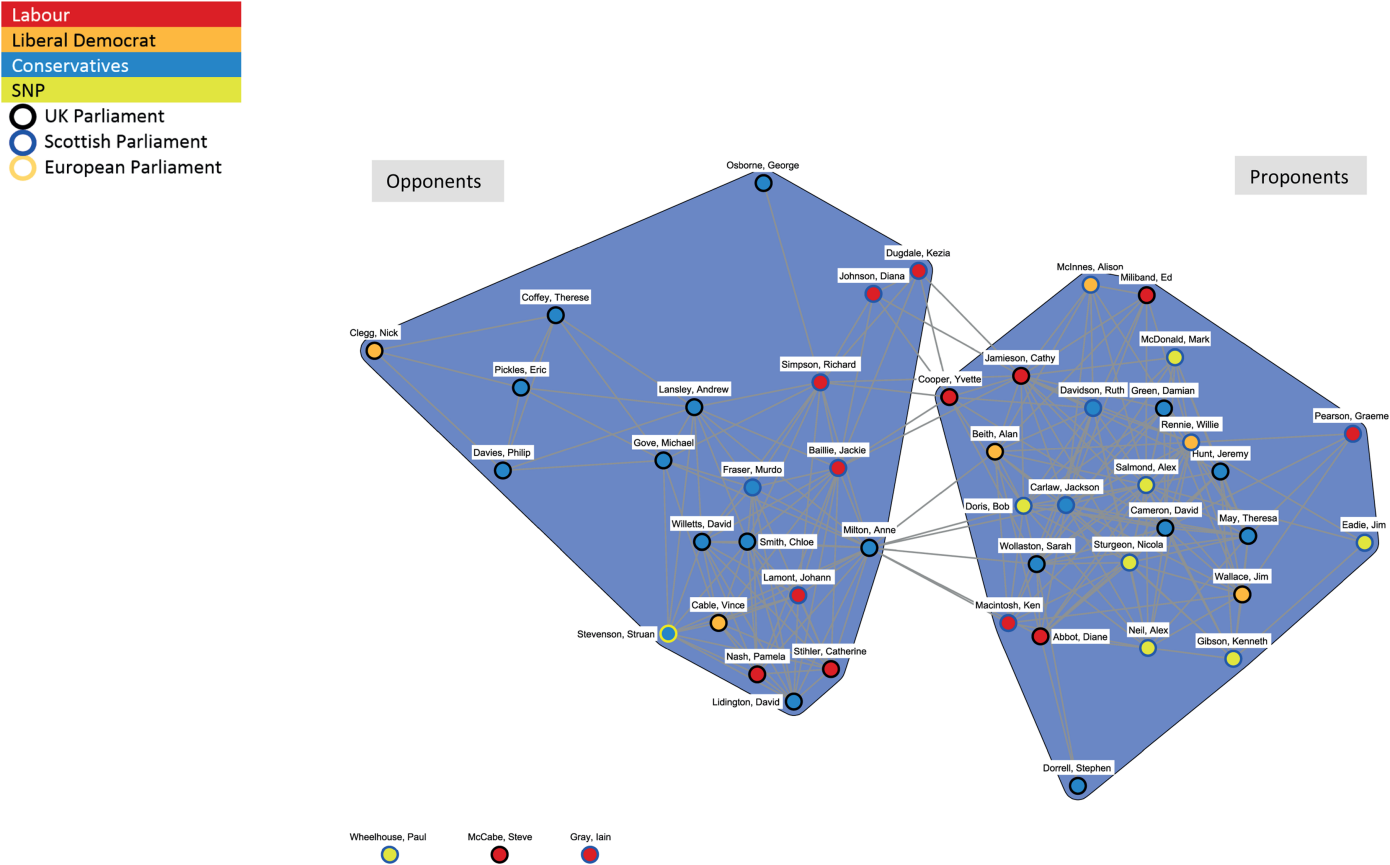
Discourse network for minimum unit pricing (MUP) (individual politicians only)

Figure 8 shows the opposing positions taken up by prominent Conservative UK Government politicians in the debate on MUP. David Cameron (Prime Minister), Theresa May (Home Office) and Jeremy Hunt (Culture, Media and Sport, then Health) are all central actors in the Proponents coalition. Andrew Lansley (Health), Michael Gove (Education) and David Willets (Universities) are central to the Opponents coalition. Liberal Democrat politicians are also split across the coalitions; however, the two Scottish Liberal Democrats in the network (Willie Rennie—Leader, and Alison McInnes—Health spokesperson) are both in the Proponents coalition. Labour politicians are also split, with two in the Opponents coalition, and several in the Proponents coalition (including the leader—Ed Miliband). The majority of Scottish Labour politicians are in the Opponents coalition, reflecting their position in opposition to the SNP in the Scottish Parliament. SNP politicians in Fig. 2 are all situated in the Proponents coalition.

### Commercial organizations

Figure 1 illustrates both cooperation and divergence among industry organizations involved in the MUP debate. Prominent central nodes in the Opponents coalition are the Wine and Spirit Trade Association and the Scotch Whisky Association appearing largest, because they were most active in the coalition. The size and position of these nodes suggest their leadership within the Opponents. Among alcohol producers, Tennant's is the sole manufacturer to be positioned within the Proponents coalition, with SAB Miller, Diageo and Heineken centrally positioned within the Opponents coalition. Tennant's, however, like Carlsberg UK, is very much on the periphery of the network, having made few statements in agreement with other organizations. The Licensed Trade organizations are also dispersed between the coalitions, with the Scottish Licensed Trade Association and Greene King positioned within the Proponents coalition and JD Wetherspoon within the Opponents coalition. Retailers are also dispersed, with Tesco positioned on the periphery of the Proponents coalition and Sainsbury's, Asda and Morrisons within the Opponents, alongside the British Retailers Consortium and Scottish Grocers Federation. This dispersal suggests a lack of co‐agreement or co‐disagreement among commercial organizations in their statements on MUP.

### Think‐tanks and charity organizations

The majority of the charities included in the network, e.g. Alcohol Concern, Alcohol Focus Scotland, are positioned centrally in the Proponents coalition with no ties to organizations in the Opponents Coalition. A charity which has previously received alcohol funding, Addaction 17, is also situated in the Proponents coalition, although more peripherally. Drinkaware, which is 96% funded by major UK alcohol producers, pub operators, restaurants, major supermarkets and other retailers 24, is also situated in the Proponents coalition. However, most of its ties are to commercial organizations, albeit those which are supportive of, or ambivalent about, the idea of MUP. Certain think‐tanks (i.e. Adam Smith Institute, Centre for Economics and Business Research and Institute for Economic Affairs) are located in the Opponents coalition along with alcohol manufacturers and retail associations, suggesting consistent alignment of the positions articulated by these groups on key concepts used by opponents of MUP.

### Academic organizations

Most academic organizations in the network feature in the Proponents coalition, including the two largest academic contributors to the debate, the University of Sheffield and Newcastle University, closely aligned with advocacy groups, alcohol‐related health charities and the SNP. Two universities are positioned peripherally to the network or within the Opponents coalition, Glasgow Caledonian University and Birmingham University, respectively. In both cases this reflects academic actors with an interest in Public Health, offering critical perspectives on MUP; for example, highlighting potential unintended consequences for alcohol‐dependent individuals, such as increased drug‐taking or criminal activities (Glasgow Caledonian University, 13 December 2011, *The Times*). In addition, individual academics from Glasgow Caledonian University with expertise in Business Studies and Philosophy commented on the policy from disciplinary perspectives outside of Public Health, in one instance citing application of JS Mill's harm principle as a litmus test for the appropriateness of laws governing individuals’ behaviour (Glasgow Caledonian University, 3 December 2011, *The Sunday Times*). Expression of such perspectives creates alignment with evidence producers in economic think‐tanks, rather than Public Health academic colleagues.

### European actors

A tightly linked portion of the Opponents coalition contains those European actors whose contributions to the debate were focused on the legality of MUP. Several wine‐producing nations who entered objections, the European Union (EU) commission, the Law Society and the European Spirits Organization, as well as the Scottish Labour Party, are drawn together by general agreement that the policy was illegal or questionable.

## Discussion

In accordance with previous research on media representations of MUP, which suggests that stakeholders were represented as either advocates or critics of the policy 14, our study shows that two polarized discourse coalitions emerged during the course of the debate drawn together by co‐agreement and co‐disagreement over conceptualizations of the alcohol problem, and responses to MUP as a potential solution. By offering a visual representation of the network of UK stakeholders engaged in the MUP debate, our study illustrates how actors cluster according to stated preferences about the policy, and provides key insights into the discursive relations of political actors, industry organizations and other key stakeholders in the debate.

The political context in which MUP was conceived and debated has been cited as an important influence on the fate of the policy proposal. The election of an SNP administration, which was less well acquainted with alcohol industry actors, has been seen as initiating renewed Scottish Government engagement with lobbyists from the alcohol industry and public health on equal terms, to end a period of relative inactivity in relation to alcohol policy 8. Early enthusiasm for MUP in the Scottish context has been explained by Katikireddi and colleagues as resultant from a range of contextual influences, including desire from the SNP Government to emulate the success of the smoke‐free legislation and follow an agenda distinct to Westminster 7. The devolved administration's pursuit of MUP also seems to have been an influential factor in the UK Government's period of support for the policy 8. Political context, however, seemed also influential in its eventual abandonment. Conservative political manoeuvrings in advance of the Scottish Independence Referendum and the 2015 General Election have been cited as key determinants of the shelving of MUP at Westminster 25. These issues of political context resonate with the structure of the network produced in this study. While the UK Conservative Party is a central node within the complete MUP network with links to both the Opponents and Proponents coalitions, the SNP node is embedded centrally within the Proponents coalition. This difference in positioning between the two parties in government at the time perhaps reflects the differing political contexts and in particular the distinctive Scottish policy context, where channels of communication between policymakers and health advocates have been characterized as more open and relationships with industry representatives less well established 7, 8.

Often in policy networks, politicians and political parties are positioned centrally, operating at the interface between two opposing discourse coalitions 26, 27. In keeping with this convention, the UK Conservative Party node inhabits a central position in the MUP network. However, this does not reflect a brokering role in relation to the development of MUP, but highlights disagreement within the party. Mapping the development of alcohol policy in England and Wales, Nicholls & Greenaway point to both ‘ideological’ and ‘systemic’ tensions in the emergence of MUP 28. They cite ideological tensions within the coalition government between libertarian and paternalistic perspectives throughout the MUP debate and identify that support for MUP within the coalition seemed bound by shifts in focus between departments and actors. Figure 8, the network of politicians, illustrates these ideological tensions and departmental shifts, with a key triad of UK Conservatives embedded within the Opponents coalition. Due to the relatively short nature of the time‐period studied, further shifts in position, both within the Home Office and from the Prime Minister, are not captured in the current analysis.

Our analysis provides insights into the dispersal of alcohol manufacturers, retailers and trade associations across both discourse coalitions. Unlike analyses of tobacco policy networks which present starkly polarized coalitions, containing clusters of homogeneous actors—either tobacco manufacturers or health‐related organizations 29—the MUP network shows the distribution of industry organizations across two opposing discourse coalitions, confirming previously identified divergence among alcohol‐related industry actors on MUP. Holden and colleagues suggest the alcohol industry is not a homogeneous entity and adopts different positions on a range of policy issues, including MUP 30. Spirits producers, which are largely export‐focused but dependent on the off‐trade for domestic sales, were the most vehemently opposed to MUP. The Scotch Whisky Association, identified as symbolic and strategic leaders of the anti‐legislation campaign 8, appear centrally in the Opponents coalition. On‐trade actors, unaffected by possible prices rises and potential beneficiaries of increased off‐sale prices, were more sympathetic to the policy, reflected in the position of the Scottish Licensed Trade Association in the network. Other sectors (e.g. beer producers and supermarkets), as evident in in Fig. 1, were more divided and the positions adopted varied at company rather than sectoral level. In all cases, those commercial organizations opposed to MUP were more vehement in their stance than those in favour, reflected in the larger size of commercial Opponent nodes in the network.

The MUP network highlights the similarities which exist between the positions articulated by free market think‐tanks and industry actors opposed to the legislation (the Wine and Spirit Trade Association, the Scotch Whisky Association and Diageo) at the centre of the Opponents coalition. Hawkins & McCambridge have described previously strategic funding of certain think‐tank reports by alcohol manufacturers which were heavily cited in the lead‐up to the UK Government's 2012 alcohol strategy 18. While neither formal nor financial relationships between think‐tanks and alcohol manufacturers are identified here, the clustering evident within the Opponents network reflects consistent co‐agreement and co‐disagreement with leading industry opponents of MUP. The network also suggests that some of the charity organizations which have been identified as recipients of alcohol industry funding 17, 31 were less centrally embedded within the Opponents coalition than might have been expected. Addaction is positioned within the Proponents coalition and the largely industry‐funded Drinkaware appears in a relatively neutral position on the periphery of the Proponents coalition, although with most links to industry organizations with a favourable, or potentially neutral, stance on MUP.

## Conclusion

Our analysis provides visual evidence of the network of stakeholders engaged in the debate on the policy of MUP to address UK alcohol consumption as portrayed in the newsprint media. The method of DNA shows much promise for extension to further our understandings of public health policy development. The current study draws on media coverage as the sole source of data, and as such the network produced reflects public narratives offered by actors engaged in the debate. Triangulating the method with other methods or with other data sources could further increase the scope of similar studies in future. Currently, our visualizations do not highlight how the positions of actors evolved beyond November 2012. We cannot provide MUP network insights on the observations of other commentators that key political events, such as preparation for the 2015 General Election or the economic concerns of the Regulatory Policy Committee, were instrumental in the UK Government's abandonment of MUP 25. The temporal focus also prohibits exploration of stakeholders’ perspectives during the period of legal challenges by the Scotch Whisky Association ending in November 2017. Future research could employ theoretically informed time–series analysis over longer periods to highlight how shifts in influential actors’ rhetoric, and positions, precede or follow key announcements about policy direction.

Illustration of co‐agreement and co‐disagreement between actors in favour and against MUP has confirmed existing evidence on the development of MUP policy in relation to political context and provided empirical evidence of some limitations of industry influence, particularly in relation to charity organizations. The findings also suggest sites for targeted public health advocacy, and confirm the potential of applying the method of DNA to contemporary policy debates in order to support identification of policy actors and organizations with an appetite for dialogue with public health organizations and active engagement with sources of public health evidence.

## Declaration of interests

None.

## Supporting information


**Data S1** List of publications included in the sample.
**Data S2** List of actor types, colour codes, actors (organisations) and acronyms.
**Data S3** List of concepts.
**Data S4** Discourse Networks by Publication.
