# Supplementary material for: Mapping discourse coalitions in the minimum unit pricing for alcohol debate: a discourse network analysis of UK newspaper coverage
Source: Addiction. 2019 Jan 4;114(4):741–53. doi: 10.1111/add.14514 (PMC6492293; doi:10.1111/add.14514)
Supplement: Supplementary file 1 — Data S1 List of publications included in the sample. Data S2 List of actor types, colour codes, actors (organisations) and acronyms. Data S3 List of concepts. Data S4 Discourse Networks by Publication. [file ADD-114-741-s001.docx]

**S1. List of publications included in the sample**

1. The Guardian / The Observer

2. Independent

3. Times / Sunday Times

4. Daily Telegraph / Sunday Telegraph

5. Daily Mail / Mail on Sunday

6. Express / Sunday Express

7. Sun / News of the World

8. Mirror / Sunday Mirror

9. Daily Record / Sunday Mail

10. Scotsman / Scotland on Sunday

11. Herald / Sunday Herald

**S2. List of actor types, colour codes, actors (organisations) and acronyms**

| Type | | Actor | Acronym |
| --- | --- | --- | --- |
| Think tank |  | Adam Smith Institute  Centre for Economic Business Research  Institute for Economic Affairs  Institute for Fiscal Studies | CEBR  IFS |
| Charity |  | Addaction  Alcohol Concern  Alcohol Focus Scotland  Alcohol Research UK  Balance  British Liver Trust  Drinkaware  Glasgow Council on Alcohol  King's Fund  Thames Reach |  |
| Supermarket |  | Asda  Morrisons  Sainsbury’s  Tesco |  |
| Professional association |  | British Medical Association  Law Society  Royal College of Nursing  Royal College of Physicians  UK Faculty of Public Health | BMA  RCN  RCP |
| Advocacy group |  | Alcohol Health Alliance  Institute of Alcohol Studies  Scottish Health Action on Alcohol Problems |  |
| University |  | Birmingham University  Centre for Addictions Research, University of Victoria  Curtin University  Glasgow Caledonian University  Newcastle University  University of Bedfordshire  University of Sheffield |  |
| Licensed trader/association |  | British Beer and Pub Association  Greene King  JD Wetherspoon  Marston's  Mitchells & Butlers  Punch Taverns  Scottish Beer and Pub Association  Scottish Licensed Trade Association |  |
| Retail association |  | British Retail Consortium  Scottish Grocers Federation  Scottish Retail Consortium |  |
| EU member state or EU body |  | Bulgaria  European Commission  France  Italy  Portugal  Spain |  |
| Government advisory body |  | Chief Medical Officer  Commons Health Select Committee  Local Government Association  Office for National Statistics  Office of Fair Trading | CMO  ONS |
| Alcohol manufacturer/  association |  | Carlsberg UK  Chivas Brothers  Comite Vins  Diageo  European Spirits Organisation  Heineken  Molson Coors  Portman Group  SAB Miller  Scotch Whisky Association  Tennent's  Wine and Spirit Trade Association | SWA  WSTA |
| Political party |  | Conservatives  Labour  Liberal Democrats  Scottish Conservatives  Scottish Labour  Scottish Liberal Democrats  Scottish National Party | SNP |
| Government |  | Scottish Government  UK Government |  |
| Government department |  | UK Government Dept for Business  UK Government Dept of Health  UK Government HM Treasury  UK Government Home Office |  |
| Consumer group |  | Campaign for Real Ale  TaxPayers Alliance |  |
| Economic consultancy/Financial organisation |  | Compecon  NCB (stockbrokers) |  |
| NHS |  | NHS Health Scotland  UK National Health Service |  |
| Police |  | Police |  |

**S3. List of concepts**

Alcohol consumption is bad for public health

Alcohol consumption is/can be bad for society

Alcohol costs NHS/government

Alcohol is increasingly marketed to young people

Alcohol misuse should be addressed by education and information

Alcohol misuse should be addressed by targetted measures

Alcohol policy can save NHS resources

Alcohol problem too complex for MUP

Alcohol related harm is falling or stabilising

Cheap, easily available alcohol drives alcohol-related health harms

Current alcohol policy measures are sufficient

Government action is needed on alcohol consumption

Government action is needed on binge drinking

Government action on public health is unnecessary

Heavy/binge/youth drinkers are price sensitive

Industry puts profits over public health

Industry should be limited to protect society

Low-income groups suffer greatest alcohol harms

MUP builds on previous tobacco and alcohol legislation

MUP could create a windfall for supermarkets

MUP could generate business for north of England

MUP does not provide positive culture change in relation to alcohol

MUP is an inappropriate intervention in the market

MUP is an unfair cost to consumer

MUP is illegal

MUP is opposed by the public

MUP is supported by evidence

MUP needed to address alcohol problem

MUP needs to be part of a package of measures

MUP needs to be set high enough to be effective

MUP should generate revenue for government

MUP will address youth drinking

MUP will be bad for business

MUP will be save public money

MUP will benefit economy

MUP will damage European industry interests

MUP will have unintended harmful consequences

MUP will increase illegal trade of alcohol

MUP will penalise responsible drinkers

MUP will protect licensed trade

MUP will reduce availability of cheapest alcohol

MUP will reduce binge drinking

MUP will reduce crime

MUP will reduce drinking

MUP will reduce foetal health harms

MUP will reduce health harms/deaths

MUP will reduce heavy drinking

MUP will reduce social problems

MUP will save public money

Majority drink responsibly

Other pricing policies are preferable to MUP

Population level measures are ineffective

Population/country has a problematic relationship with alcohol

Problematic alcohol consumption driven by social and economic conditions

Responsibility deals with alcohol industry are ineffective

| Think tank |
| --- |
| Charity |
| Supermarket |
| Professional association |
| Advocacy group |
| University |
| Licensed traders and associations |
| Retail associations |
| EU member state or EU body |
| Government advisory body |
| Alcohol manufacturers |
| Political party |
| Government |
| Government department |
| Consumer group |
| Economic consultancy |
| NHS |
| Police |

**S4. Discourse Networks by Publication**
